# Neural structures associated with auditory short-term memory and sentence repetition following chronic left hemisphere stroke

**DOI:** 10.3389/fnins.2026.1867156

**Published:** 2026-08-20

**Authors:** Yuexin Mei, Huijia Tang, Yalin Yuan, Shuhan Fan, Xingyang Niu, Xinyan Fan, Jinsheng Zeng, Shihui Xing

**Affiliations:** 1 Department of Neurology, The First Affiliated Hospital, Sun Yat-sen University, Guangzhou, China; 2 Guangdong Provincial Key Laboratory of Diagnosis and Treatment of Major Neurological Diseases, National Key Clinical Department and Key Discipline of Neurology, Guangzhou, China

**Keywords:** aphasia, arcuate fasciculus, auditory short-term memory, repetition, stroke

## Abstract

**Introduction:**

Prior evidence suggests auditory short-term memory (STM) and repetition share several processing elements. However, it remains elusive whether auditory STM capacity and repetition ability show overlapping neural correlates. We sought to identify the brain structures underpinning STM capacity and repetition outcomes in patients with chronic left hemisphere stroke and aphasia.

**Methods:**

Fifty-four left hemisphere stroke patients with aphasia and 50 demographically matched healthy controls underwent digit span and language tasks. Structural and diffusion images were acquired in all participants. Support vector regression-based lesion-symptom mapping (SVR-LSM) analyses were conducted to identify critical brain regions associated with auditory STM capacity, as indexed by transformed digit span scores and word/sentence repetition performance. The relationships between direct and indirect arcuate fasciculus (AF) injury and behavioral performance were examined.

**Results:**

Sentence repetition was associated with digit span performance by regressing out confounding variables. SVR-LSM identified the left parietal lobe, including the supramarginal gyrus and inferior parietal cortex, and the corresponding white matter regions were correlated with auditory STM capacity and sentence repetition after adjusting for control variables. Additionally, the direct lesional damage or the integrity of the left AF was associated with auditory STM and sentence repetition independent of the lesion load on the gray matter. When controlling for the damage of the left AF, the correlation with the left inferior parietal cortex with auditory STM capacity remained significant.

**Conclusion:**

Our findings suggest that the AF, along with the inferior parietal cortex in the left hemisphere, is associated with auditory STM capacity and sentence repetition.

## 1 Introduction

Working memory plays an important role in language processing by temporarily storing and maintaining verbal information during discourse. How working memory supports language processing is still a matter of debate. The classic model of working memory proposed by Baddeley and Hitch indicates three components, including central control, phonological loop, and visuospatial sketchpad, representing temporary information storage and manipulation of complex cognitive performance (Baddeley, 1992, 2010). According to Cowan’s conceptualization, working memory includes short-term memory (STM) and the attentional control mechanisms that support its use (Cowan, 2008). In contrast to the broader working memory construct that entails both storage and concurrent processing, short-term memory (STM) refers specifically to the passive temporary maintenance of information (Baddeley, 2003b). As a matter of fact, the relationship between STM and language processing has been suggested (Martin et al., 2018; Varkanitsa and Caplan, 2018; Geva et al., 2021). Phonological deficits are commonly observed in aphasic patients, which may be attributed to damage to the phonological loop (Baddeley, 2003a). In natural language processing, the development of phonological working memory impacts language learning (Delcenserie et al., 2021; Juffs and Harrington, 2011). As such, linguistic storage may be one basic and automatic element of STM tasks, as evidenced by the studies showing that the activation of stored linguistic knowledge requires executive control (Hoffman et al., 2012). Among different processes of language, speech comprehension is of great importance (Daneman and Merikle, 1996), and a rapid processing framework is required for understanding and maintaining the memory system when hearing a sentence (Lewis et al., 2006).

Repetition processing involves two separate elements involving reproduction and storage of information, which are likely supported by STM capacity. The interactive model of word processing suggests that deficits in short-term activation maintenance would affect word repetition ability (Dell et al., 2007), whereas repetition-based treatment can enhance word processing ability in aphasic patients (Martin et al., 2021). In contrast to word repetition, sentence repetition is predominantly manipulated by phonological memory capacity (Andreou et al., 2020). Patients with acquired impairment of auditory STM commonly demonstrated sentence repetition deficits (Warrington and Shallice, 1969; Peter, 2018). The ability to repeat long sentences or syllables might be influenced by impairment of STM (Sakurai et al., 1998). Collectively, these findings indicate that brain regions associated with auditory STM might be a subset of those supporting sentence repetition.

Evidence from lesion-symptom map studies have shown the left temporo-parietal cortex to be critical for repetition and auditory STM independent of the arcuate fasciculus (AF) (Baldo et al., 2012). Counter to the traditional disconnection model emphasizing the AF, a revised language framework for repetition highlights the indirect pathway connecting Broca’s and Wernicke’s areas and the left temporal–parietal cortex (Forkel et al., 2020). Crucially, cases with AF injuries after cerebral infarcts, tumors, or lesions beyond AF boundaries could directly or indirectly affect adjacent bundles or cortices in patients exhibiting repetition deficits (Yamada et al., 2007; Chernoff et al., 2020). Intracranial stimulation studies revealed that stimulation of cortical areas, including the posterior portion of the left superior temporal gyrus (STG) and the supramarginal gyrus (SMG), can also induce impaired repetition despite intact comprehension (Quigg et al., 2006). However, the associations between the AF and repetition had not been determined, and whether the AF is necessary to support repetition and auditory STM in patients with chronic post-stroke aphasia needs to be elucidated.

Auditory STM is commonly measured by digit span in healthy subjects (Wechsler, 1997). In natural language, digit span appears to be involved in random sequences, which supports the STM ability (Jones and Macken, 2015). However, the inter-individual behavioral variability might be a result of different cognitive items that influence STM performance (Leff et al., 2009). Accumulating evidence revealed an intrinsic association of STM with speech comprehension and production in patients with post-stroke aphasia (Minkina et al., 2018). Additionally, individuals with post-stroke aphasia might perform below normal levels during auditory STM tasks. Thus, it is necessary to eliminate those components that might influence the accuracy of auditory STM assessment.

Here, we used support vector regression-based lesion symptom mapping (SVR-LSM) to identify the critical neural structures supporting auditory STM and to determine whether these regions also supported sentence repetition in patients with chronic post-stroke aphasia. Additionally, we explored the roles of AF in sentence repetition and auditory STM using tract-based analysis. We hypothesized that gray matter or white matter supporting auditory STM might be associated with sentence repetition ability in post-stroke aphasia.

## 2 Methods

### 2.1 Participants

Fifty-four patients (17 female) who had suffered a first left hemisphere stroke with a history of aphasia were recruited with the following inclusion criteria: native Chinese speakers at least 6-month post-stroke; able to follow testing instructions; no history of prior neurological or psychiatric disorders or substance abuse. All patients had aphasia at the time of stroke based on medical records. Additionally, 50 demographically matched healthy subjects (21 female) without any history of neurological or psychiatric disorders were recruited in the study as controls. See Table 1 for characteristics of the two groups. The study was approved by the Institutional Review Board of the First Affiliated Hospital of Sun Yat-sen University, and written informed consent was obtained from all participants before enrolment.

**Table 1:** Demographic details and behavioral test scores in patients and healthy subjects.

	Patients (*n* = 54)	Controls (*n* = 50)	Statistics	*p*-value
Demographic variable				
Age (years)	54.92 (11.91)	52.29 (12.81)	t(102) = 1.09^1^	0.26
Gender (M/F)	37/17	29/21	χ2(1) = 1.24	0.27
Education (years)	10.63 (3.67)	11.67 (4.32)	t(102) = −1.33^1^	0.19
Handedness (R/A)	53/1	47/3	χ2(1) = 1.21	0.27
Time post stroke (months)	24.38 (18.64)	–	–	–
Lesion size (cm3)	64.80 (77.25)	–	–	–
Behavioral tests				
Forward digit span	8.50 (4.59)	13.36 (2.02)	t(102) = −7.08^1^	<0.001
WAB aphasia quotient	76.9 (23.1)	98.90 (1.19)	t(102) = −7.01^1^	<0.001
Auditory-verbal comprehension	8.20 (1.93)	9.91 (0.01)	t(53) = −6.53^2^	<0.001
Word fluency	8.11 (5.44)	18.42 (4.49)	t(102) = −12.92^1^	<0.001
Auditory word repetition	30.78 (7.01)	34.00 (0.00)	t(53) = −3.38^2^	0.001
Auditory sentence repetition	52.83 (19.68)	65.76 (0.72)	t(53) = −4.82^2^	<0.001

Parentheses indicate standard deviations. ^1^ Student’s *t*-test, ^2^ Welch’s *t*-test. M, male; F, female; n, number; R/A, right/ambidextrous handedness. WAB, Western-Aphasia Battery. Normal range: WAB aphasia quotient, 0–100; forward digit span, 0–16; auditory-verbal comprehension, 0–10; word fluency, 0–20; word repetition, 0–34; auditory sentence repetition, 0–66.

### 2.2 Behavioral assessments

#### 2.2.1 Digit span task

The forward digit span task was applied to examine the capacity of auditory STM according to standard procedures (Wechsler, 1997). We scored the number of items repeated correctly, with the maximum score of 16. Given speech output disorders in aphasia, participants were asked to complete a point span task after their failure, which used the same order as the next-length item without the last digit. They were instructed to point to the same digits on the written number line after an auditory presentation, beginning at the level where they reached ceiling on the digit span task. It had been shown that verbal span would not vary depending upon the paradigm (pointing response versus repetition) used to assess span in aphasic patients (Martin and Ayala, 2004).

#### 2.2.2 Language tasks

All participants were administered the Western Aphasia Battery-Revised (WAB-R) (Kertesz et al., 1982), which was translated into Chinese and includes subtests that provide composite scores for spontaneous speech, repetition, naming/word-finding, and auditory verbal comprehension. The language assessment was conducted and scored by two neurologists. The repetition task included nine items of word repetition (syllable range 1–5) and six items of sentence repetition. The recognizable syllable or word repetition corresponds to repeating the single digit during digit span test, in which the capacity of auditory STM is not stretched. In contrast, sentence repetition should require higher memory load to a certain degree, and is more likely influenced by auditory STM capacity. To identify the capacity of auditory STM as much as possible, cognitive components required for the digit span task were controlled as previously reported (Leff et al., 2009). Auditory verbal comprehension was used to control word- or sentence-level speech perception and comprehension during digit-span processing. Word fluency derived from the naming/word-finding subtest was applied to control executive processes in digit span performance. In parallel with digit span, auditory word and sentence repetition were also taken into account.

### 2.3 Image data acquisition

Image data were acquired on a 3 T Siemens Trio scanner at the Cancer Centre of Sun Yat-sen University. The structural images were acquired using a 3D high-resolution T1-weighted sequence (magnetization-prepared rapid-acquisition gradient echo, MPRAGE) with the following parameters: repetition time (TR) = 1900 ms; echo time (TE) = 2.56 ms; flip angle = 9°; field of view (FOV) = 250 × 250 mm^2^; matrix size = 246 × 256; voxel size = 1 × 1 × 1 mm^3^; 192 contiguous 1 mm sagittal slices; slice thickness = 1 mm. Diffusion-weighted imaging (DWI) was acquired using a single-shot echo-planar imaging sequence with the following parameters: TR = 8700 ms; TE = 90 ms; flip angle = 90°; FOV = 240 × 240 mm^2^; matrix size = 96 × 96; voxel size = 2 × 2 × 3 mm^3^; slice thickness = 3 mm; 64 diffusion volumes weighted with b_max_ of 1100 s/mm^2^ and one volume weighted with no diffusion gradient (b_0_ = 0 s/mm^2^).

### 2.4 Data preprocessing

#### 2.4.1 Behavioral data preprocessing

The ability of digit span might be strongly affected by demographic factors (e.g., age, gender and education), with considerable variability. Raw scores of digit span might not meaningfully reflect the degree of deficits among patients. As previously described (Crawford and Garthwaite, 2006), the raw score of digit span was corrected and transformed into a standardized *t*-score for individual patients by accounting for the performance distribution in healthy controls. A regression model based on the control group was established with the raw score of digit span as the dependent variable and the demographic factors, including age, gender and education, as the predictors. The demographic information of each patient was introduced into the model to acquire the predicted accuracy score, and a discrepancy value (*Discrepancy*_patient_) was generated (observed accuracy-predicted accuracy). The following formula was used to acquire the corrected standard error of estimate for each patient (*SE*_patient_) as previously reported (Crawford and Garthwaite, 2006):

$SE_{\text{patient}=S_{Y\cdot X}}\sqrt{1+\frac{1}{N}+\frac{1}{N-1}\sum r^{ii}Z_{\mathrm{io}}^{2}+\frac{2}{N-1}\sum r^{ij}Z_{\mathrm{io}}Z_{\mathrm{jo}}}$

where *S_Y·X_* and *N* are the standard error and sample size for the control group; *r^ii^* and *r^ij^* the main diagonal and off-diagonal elements of the inverted correlation matrix for the *k* predictor variables (*k* = 3); and *z_O_* (*z_1O_*,.., *z_kO_*) is the *z*-scores of the accuracy of the patient on the predictor variables. The *t*-score for each patient was computed using the following formula: *t-score*_patient_ = *Discrepancy*_patient_/*SE*_patient_. The raw data of language performance were not transformed as above because a potential ceiling effect might be found in healthy participants (Leff et al., 2009).

#### 2.4.2 Imaging data preprocessing

##### 2.4.2.1 Lesion mapping protocol

**Figure 1:**
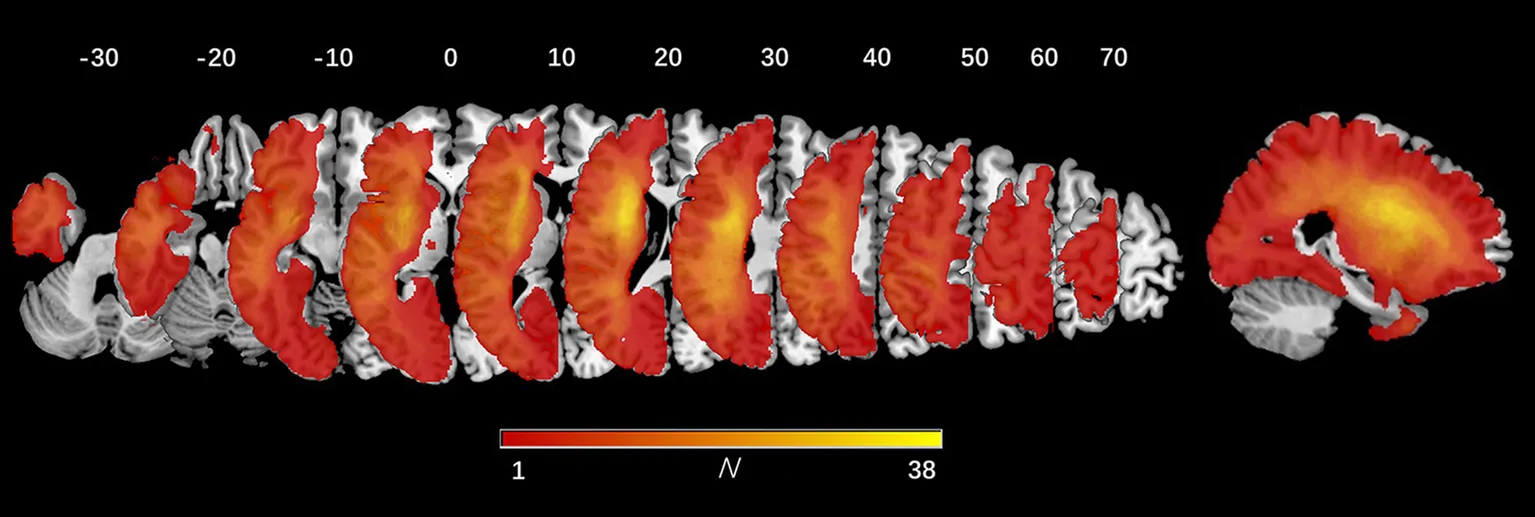
Lesion overlap map of the patient cohort. Lesion distribution is shown for 54 patients with chronic post-stroke aphasia. Lesions were manually delineated on individual T1-weighted imaging and subsequently warped into Montreal Neurological Institute space. The color bar indicates the number of overlapping lesions at each voxel. Red on the scale indicates a lower number of subjects, and yellow indicates a higher number (maximum 38 out of 54).

Stroke lesions were manually drawn on the T1-weighted images in the respective native space using MRIcron software (Rorden and Brett, 2000)^1^ and independently confirmed by two neurologists blinded to the participants’ language scores. The mean Dice coefficient was 0.93 ± 0.09, indicating an excellent agreement between raters. The raw lesion maps were then normalized to the Montreal Neurological Institute (MNI) space to allow direct comparisons across individuals, using the enantiomorphic approach with minor modifications by conducting the unified segmentation toolbox built in CAT12^2^ under SPM12 (Nachev et al., 2008). The lesion overlap map of 54 patients is shown in Figure 1.

##### 2.4.2.2 Diffusion data preprocessing

Diffusion-weighted images were pre-processed using the FMRIB software library (FSL^3^), including skull stripping, eddy current correction, tensor fitting, and reorientation. Spatial normalization was performed using a non-parametric, diffeomorphic deformable image registration technique with diffusion tensor imaging (DTI) - ToolKit, which is optimized for examining white matter morphometry based on higher-order information of DTI^4^. FA images of each dataset were obtained from the respective spatially normalized tensor images using the transformations derived from the previous alignments, with a threshold of 0.2 to exclude spurious voxels (Zhang et al., 2006).

### 2.5 Brain structure-behavioral correlates

#### 2.5.1 Support vector regression-based lesion-symptom mapping analysis

Support vector regression-based lesion-symptom mapping (SVR-LSM), a multivariate lesion-symptom mapping approach, was used to examine critical subcortical areas in the left hemisphere in which damage relates to behavioral performance as previously described (DeMarco and Turkeltaub, 2018). Only voxels damaged in at least 10% of patients were included in the analysis. Probabilistic maps were created using 10,000 permutations of the behavioral scores with a voxel-wise cluster-forming threshold of *p* < 0.005 and cluster-level family-wise error correction at *p* < 0.05, restricted to the left hemisphere mask. We first identified the crucial brain regions to support *t* score of digit span (*t*-DS) entered as the dependent variable by controlling for lesion size. To explore the brain regions supporting STM capacity, a separate SVR-LSM analysis was conducted with *t*-DS as the dependent variable, including word fluency, auditory verbal comprehension, and word or sentence repetition as control variables in a stepwise manner.

#### 2.5.2 Tract-symptom correlation analysis

Based on SVR-LSM results, we next explored the relationship between the AF tract and behavioral performance. The left AF was reconstructed from healthy participants. In brief, the corresponding paired regions of interest (ROIs) in the left hemisphere were defined as previously described (Wakana et al., 2007), and then transformed back to the subject’s native DWI space using non-linear registrations for FA images. Fiber tracking was performed using a probabilistic tractography algorithm implemented in the FSL toolbox (probtrackx), based on Bayesian estimation of diffusion parameters (Bedpostx) (Behrens et al., 2007). From each mask voxel, 5,000 streamline samples with a step length of 0.5 mm and a curvature threshold of 0.2 were generated to map the probabilistic connection pattern. The tract density map was obtained by dividing by the total number of streamline samples, and then thresholded at 1% to exclude spurious connections as suggested in previous studies (Ding et al., 2024; Roth et al., 2024; Wilmskoetter et al., 2022). The resultant density maps were normalized to MNI space. The final group-wise probabilistic map of the left AF was generated by summing the thresholded density maps across healthy subjects and thresholded at a level of 25%, indicating that over 12 of 50 participants consistently had the connections within each voxel of the tract. To verify the robustness of the tract-behavioral correlation results, the group-wise probabilistic AF template was additionally created at an alternative threshold of 50%.

To explore the contributions of the left AF tract to patients’ performances, we firstly evaluated the associations of stroke lesion load on the left AF (referred to as direct damage) with patients’ scores on t-DS and sentence repetition. The stroke lesion percentage on the left AF was obtained from the number of lesioned voxels registered to the same space as the tract map divided by the total number of tract voxels. Additionally, to explore whether FA values of the tract reveal information beyond the direct effect of the lesion (referred to as indirect damage), we calculated the partial correlation between the mean FA values of the left AF and patients’ behavioral performance scores.

### 2.6 Statistical analysis

Demographic and behavioral data between groups were compared using Student’s *t*-tests, Welch’s *t*-tests or chi-square tests, where appropriate. To assess tract–behavior associations, we performed partial correlations between the direct (lesion load) and indirect (FA values) damage indexes of the left AF and the t-DS score, controlling for lesion size, chronicity, and behavioral control variables. Additionally, partial correlations between the respective damage indices of the left AF and sentence repetition scores were conducted by controlling for age, gender, level of education, chronicity, and lesion size. For each of these partial analyses, multiple comparisons were corrected at a threshold of *q* < 0.05 with false discovery rate (FDR). The full simultaneous regression was conducted to examine the potential correlates of t-DS performance, including auditory verbal comprehension, word fluency, word repetition, and sentence repetition. The standardized beta coefficients (β) for each predictor, variance inflation factor (VIF), and the overall model fit were reported. A *p*-value of <0.05 was considered statistically significant. Additionally, a dominance analysis was performed to identify the potential predictors of t-DS performance. Statistical analyses were performed using SPSS (version 26) or R software (version 4.3.0).

## 3 Results

### 3.1 Demographic and behavioral results

Demographic details and behavioral scores are shown in Table 1. There were no significant differences in age, gender, level of education, or handedness between patient and healthy control groups. The mean aphasia quotient (AQ) in patients was 76.9 ± 23.1. Aphasia types included anomic aphasia (*n* = 27), recovery (*n* = 13), Broca’s aphasia (*n* = 7), Wernicke’s aphasia (*n* = 2), conduction aphasia (*n* = 2), global aphasia (*n* = 1), transcortical sensory aphasia (*n* = 1), and transcortical motor aphasia (*n* = 1). As expected, healthy controls performed at ceiling on auditory word repetition, auditory verbal comprehension, and sentence repetition tests. Compared with the controls, patients had significant decreases in the raw digit span, AQ, word/sentence repetition, auditory verbal comprehension, and verbal fluency scores (*p* < 0.001). The normalized *t*-DS for patients was −15.51 ± 14.72, obtained from a regression model built on the healthy group with demographic characteristics as covariates. When controlling for lesion size, *t*-DS score was positively correlated with scores on auditory verbal comprehension (*r* = 0.46, *p* < 0.001), word fluency (*r* = 0.33, *p* = 0.015), word repetition (*r* = 0.56, *p* < 0.001), and auditory sentence repetition (*r* = 0.70, *p* < 0.001). Subsequent full simultaneous regression analysis showed that the *t*-DS performance was significantly associated with the scores of auditory sentence repetition (*β* = 0.948, VIF = 8.18, *p* < 0.001) but not auditory-verbal comprehension (*β* = −0.036, VIF = 5.27, *p* = 0.846), word fluency (*β* = 0.055, VIF = 2.55, *p =* 0.669), or word repetition (*β* = −0.149, VIF = 4.53, *p* = 0.389). The model accounted for 68.4% of the variance [adjusted *R*^2^ = 0.658, *F*(4,49) = 26.46, *p* < 0.001]. Additionally, the dominance analysis showed that sentence repetition (dominance weight = 0.294, 43.0% of *R*^2^) was mostly correlated with *t*-DS score relative to auditory-verbal comprehension (dominance weight = 0.152, 22.2% of *R*^2^), word fluency (dominance weight = 0.089, 13.0% of *R*^2^), and word repetition (dominance weight = 0.149, 21.8% of *R*^2^). Collectively, these results indicate a potential correlate between sentence repetition and STM performance.

### 3.2 Identification of critical left hemisphere areas related to repetition and auditory STM

**Figure 2:**
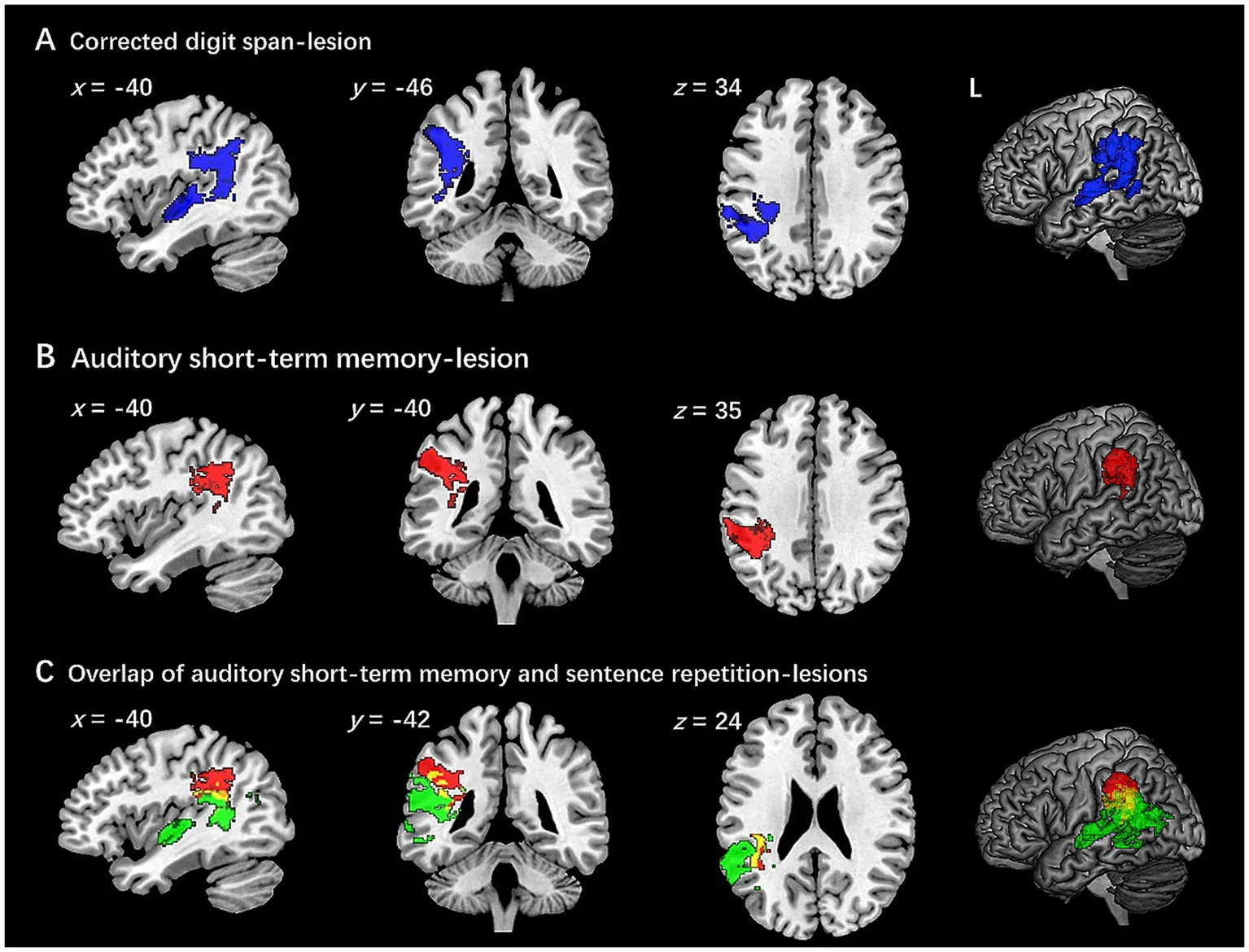
Lesion-behavior maps of auditory-verbal short-term memory and sentence repetition identified by multivariate support vector regression-based lesion-symptom mapping (SVR-LSM). SVR-LSM results illustrating the critical brain regions associated with behavioral performance, thresholded at voxel-wise *p* < 0.005 and cluster-level family-wise error correction at *p* < 0.05 within the left hemisphere mask. **(A)** Regions associated with *t* scores of digit span (*t*-DS) located in the left posterior perisylvian area, encompassing the superior and middle temporal gyri, supramarginal gyrus (SMG), inferior parietal lobule (IPL), and the corresponding white matter. **(B)** When controlling for auditory verbal comprehension, word fluency, and word repetition, regions specific to auditory short-term memory were constrained to the left SMG, IPL, and the subcortical white matter. **(C)** Overlapped region (yellow) of SVR-LSM maps on sentence repetition (green) and auditory short-term memory (red), involving the left SMG, IPL, and the underlying white matter tracts. L, left.

The relationships between lesion location in the left hemisphere and corrected digit span (*t*-DS) scores were examined using SVR-LSM accounting for individual stroke lesion sizes. The SVR-LSM analysis revealed the critical brain regions associated with the *t*-DS score in the left posterior perisylvian area including the left STG and middle temporal gyrus (MTG), SMG, and inferior parietal lobule (IPL), as well as their corresponding white matter (Figure 2A). To account for language domains involved in the digit span task but unrelated to auditory STM capacity, we included additional behavioral variables, including auditory verbal comprehension, word fluency, and auditory word or sentence repetition, in the subsequent SVR-LSM analyses. When controlling for auditory verbal comprehension, word fluency, and auditory word repetition scores, the SVR-LSM analysis of the *t*-DS score identified critical regions in the left SMG and IPL, along with subcortical white matter (Figure 2B). When the auditory sentence repetition score was further included, no significant voxels were identified in the SVR-LSM analysis on *t*-DS score. Given the strong correlation between sentence repetition and auditory STM, we explored potential common areas between these two processes. Next, we conducted additional SVR-LSM analysis on sentence repetition score by factoring out stroke lesion sizes. The results showed that there were considerable overlapping lesion locations in the left hemisphere associated with auditory STM capacity and sentence repetition, including the left SMG, IPL, and their subcortical white matter (Figure 2C). Additionally, we determined the volume proportions of critical gray and white matter identified in the SVR-LSM analysis of auditory STM capacity and sentence repetition. The results showed that 52.52% of the overlapped lesion regions were within the white matter structure and 47.48% were located in gray matter. Therefore, it is worth exploring the role of AF in sentence repetition and auditory STM by combining structural and diffusion data, as white matter structure also contributes to these two abilities.

### 3.3 Correlates of the left AF with repetition and auditory STM performance

**Figure 3:**
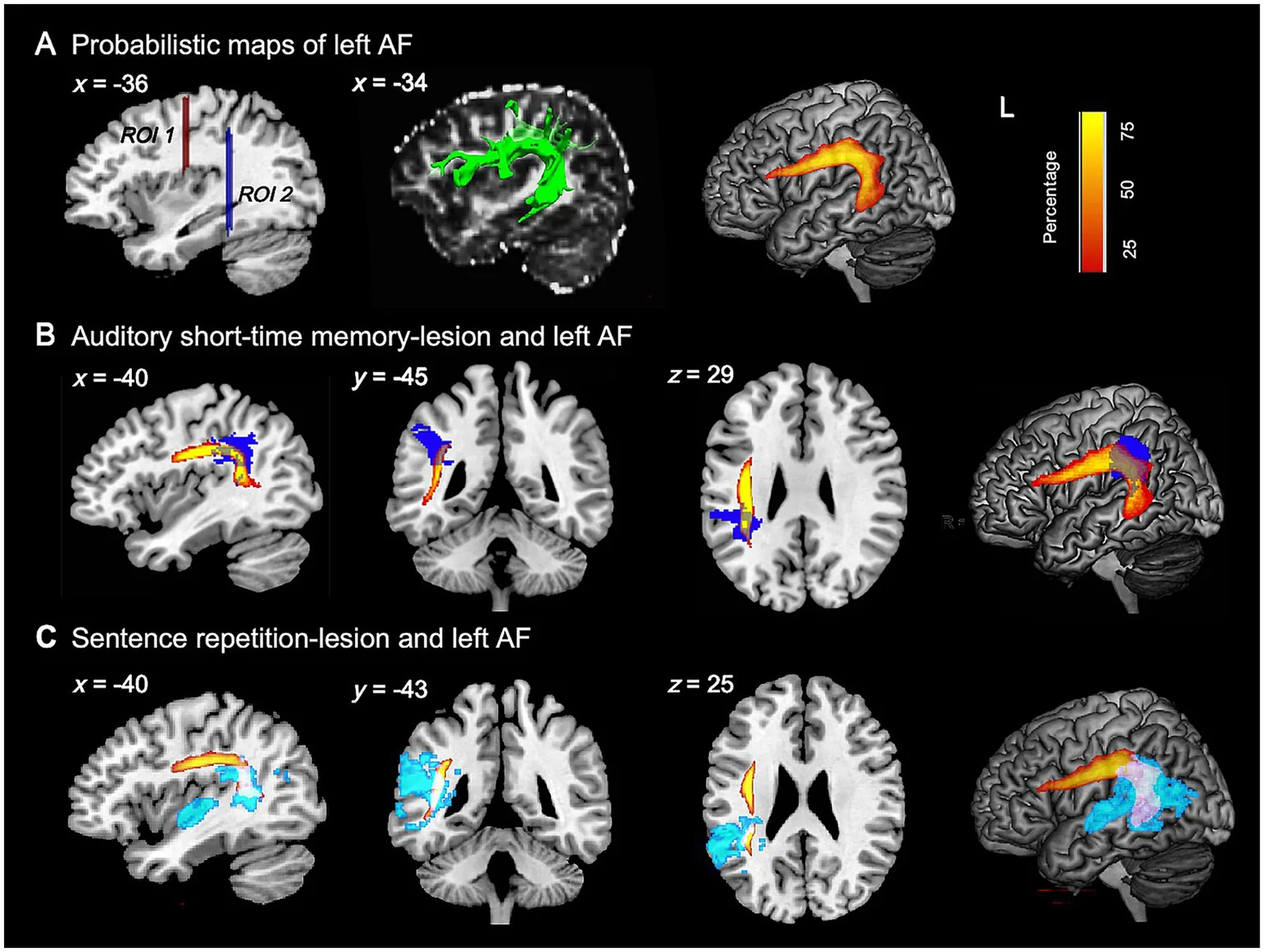
Spatial overlap between the left arcuate fasciculus and critical lesion sites. **(A)** Probabilistic tractography map of the left arcuate fasciculus (AF) reconstructed from the control group. The color bar indicates the percentage of tract overlap across participants. **(B)** Overlay of the critical lesion map for auditory short-term memory onto the left AF. The deficit-associated regions predominantly intersect the middle-posterior portion of the tract. **(C)** Overlay of the critical lesion map for sentence repetition onto the left AF, demonstrating a similar spatial distribution along the middle-posterior segment. Numbers indicating MNI coordinates (x, y, z). AF, arcuate fasciculus; L, left.

**Figure 4:**
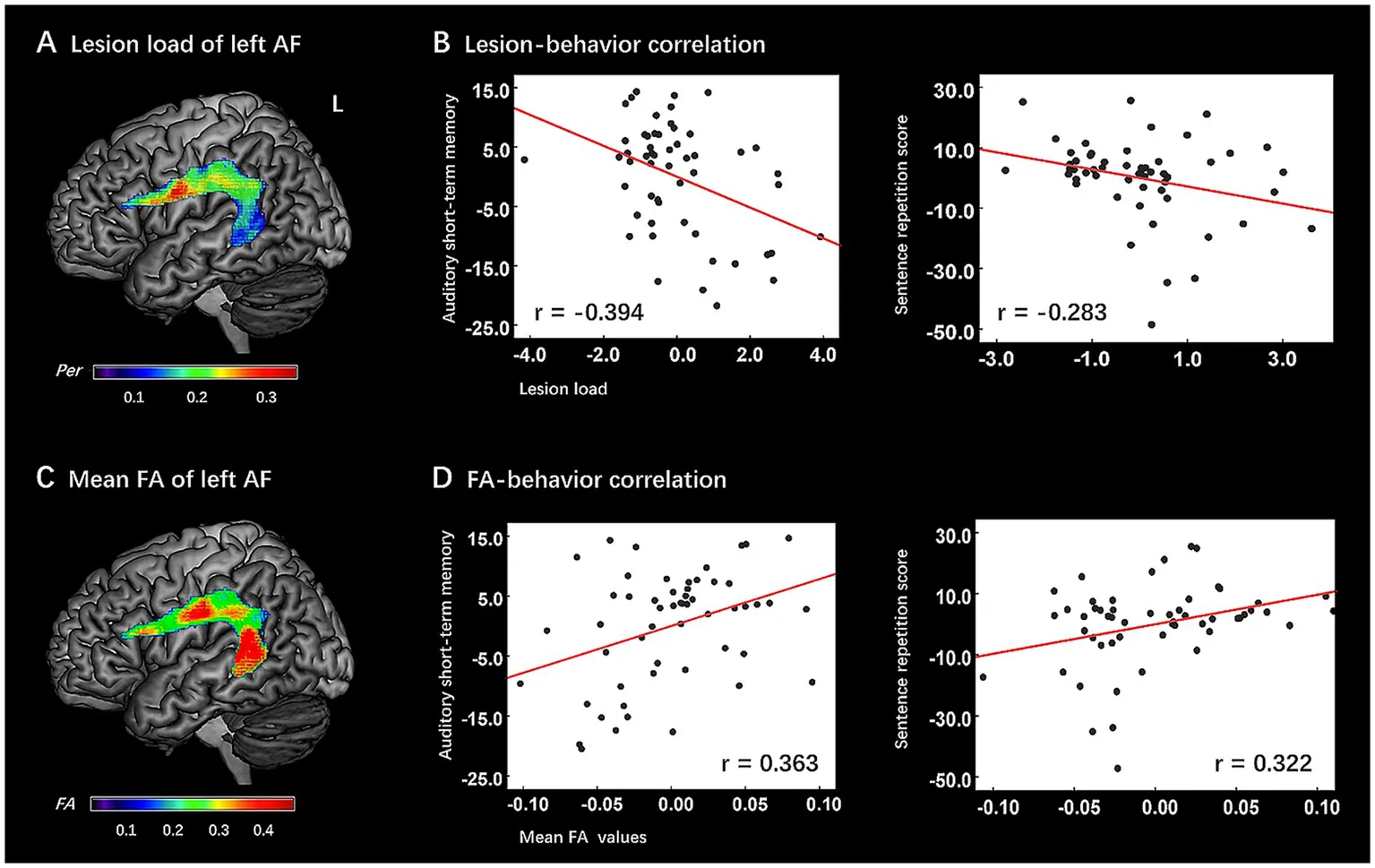
Relationships between left arcuate fasciculus structural integrity and behavioral performance. **(A)** Spatial distribution of the direct lesion load on the left arcuate fasciculus (AF), localized predominantly to the anterior and middle portions. The color bar indicates the proportion of lesion overlap. **(B)** Partial correlations demonstrate a significantly negative relationship between AF lesion load and auditory short-term memory performance after controlling for language variables, stroke volume, and chronicity (*r* = −0.394, *p* = 0.017, FDR correction) and a marginally significant association with sentence repetition (*r* = −0.283, *p* = 0.056, FDR correction) by factoring out demographic variables, stroke volume, and chronicity. **(C)** Voxel-wise distribution of mean fractional anisotropy (FA) within the left AF, demonstrating reduced microstructural integrity primarily in the anterior portion. **(D)** Scatter plots illustrate positive correlations between the mean FA values of the AF and performance in auditory short-term memory (*r* = 0.363, *p* = 0.025, FDR correction) and sentence repetition (*r* = 0.322*, p* = 0.038, FDR correction). AF, arcuate fasciculus; FA, fractional anisotropy; L, left; Per, percentage.

To define potentially relevant white matter tracts, the left AF was reconstructed in the control group (Figure 3A). We overlapped the lesion maps of the auditory STM capacity and sentence repetition identified above on the left AF, and found that the critical regions identified in the SVR-LSM analyses on auditory STM capacity or sentence repetition were evidently distributed in the middle-posterior portion of the left AF (Figures 3B,C). Subsequently, we performed lesion load-behavior analysis by extracting the individual lesion load of the left AF to examine the direct damage to the AF on behavioral performance. The direct lesion load on AF was observed to be predominantly distributed on the anterior and middle portions of the left AF (Figure 4A). After controlling for language control variables, stroke volume, and chronicity, there was a significant negative correlation between the lesion percentage of AF and *t*-DS score (*r* = −0.394, *p* = 0.017, FDR correction; Figure 4B). Additionally, a marginally negative correlation was found between the lesion load of AF and sentence repetition score after adjusting for demographic characteristics, stroke volume, and chronicity in patients (*r* = −0.283, *p* = 0.056, FDR correction; Figure 4B). With respect to tract-behavior correlation analysis, the reduced FA value of voxels was observed in the anterior portion of the left AF (Figure 4C). The mean FA values of the left AF were significantly correlated with *t*-DS score when controlling for language control variables, chronicity, and lesion volumes (*r* = 0.363, *p* = 0.025, FDR correction). For sentence repetition, the mean FA values of the left AF were significantly related to sentence repetition score when controlling for demographic factors, chronicity, and lesion volumes (*r* = 0.322, *p* = 0.038, FDR correction; Figure 4D). Additionally, we conducted the analysis using the tract template with an alternative threshold of 50% to verify the robustness of the current findings. The results remained significant regarding the correlates of lesion load or FA values of the left AF with *t*-DS score (*r* = −0.405, *p* = 0.008 and *r* = 0.408, *p* = 0.008; FDR correction) as well as sentence repetition performance (*r* = −0.290, *p* = 0.044 and *r* = 0.329, *p* = 0.028; FDR correction).

### 3.4 Unique contributions of gray/white matter to repetition and auditory STM performance

Subsequently, we explored the relative contributions of gray matter and white matter structures to sentence repetition and auditory STM performance by computing partial correlations between behavioral performance and the lesion percentage of gray matter or the mean FA value, with each as an additional covariate. The results demonstrated the t-DS score to be negatively associated with the lesion load of the left AF (*r* = −0.417, *p* = 0.004) and positively with the mean FA values (*r* = 0.288, *p* = 0.047) when factoring out language control variables, lesion size, and lesion percentage values of the left SMG or IPL. When considering damage extent of the left AF, the *t*-DS score was negatively correlated with lesion percentage of the left IPL (*r* = −0.464, *p* = 0.001), rather than lesion percentage of the left SMG (*r* = −0.240, *p* = 0.100). Besides, the correlation between mean FA values of the left AF and sentence repetition remained significant when the lesion percentage values of gray matter were additionally controlled (*r* = 0.316, *p* = 0.026). By contrast, when considering damage extent of the left AF as additional covariates, no significant correlations were found between sentence repetition score and the lesion percentage of the left IPL or SMG (*p* > 0.05). These results suggest the overweighted contribution of the left AF over gray matter to auditory STM and sentence repetition among chronic stroke patients with aphasia.

## 4 Discussion

In the present study, we found that auditory STM capacity and sentence repetition were correlated cognitive processes. The lesion-behavior analysis revealed that the damage of the left IPL was significantly related to the abilities of these two processes. Furthermore, the integrity of the left AF was negatively correlated with deficits in both behavioral performances, even after accounting for lesion load in the left hemisphere gray matter. Taken together, these results suggest that the left AF and IPL are associated with auditory STM capacity and sentence repetition after chronic left hemisphere stroke.

### 4.1 Sentence repetition and auditory verbal STM are correlated

Both our behavioral and imaging analyses supported the close correlations between auditory STM and sentence repetition. The relationship between auditory STM and sentence repetition has been suggested by Cowan’s embedded processes model, and the sentence repetition task might require concurrent activations of multiple word representations (Cowan, 1999). The auditory STM has a capacity limit, which means only a limited number of items can be activated at the same time (Chen and Cowan, 2009). The auditory STM deficits indicated a reduced number of word representations that could be activated simultaneously, leading to failure in sentence repetition.

The current observation is consistent with the previous hypothesis model of conduction aphasia (Warrington and Shallice, 1969; Shallice and Warrington, 1977). As a matter of fact, two different forms of conduction aphasia have been suggested as efferent/afferent or reproduction/repetition (Bernal and Ardila, 2009). The efferent-reproduction type involved the phonemic organization and representation of words, whereas afferent-repetition conduction aphasia involved STM deficits affecting the repetition of large strings of materials (Shallice and Warrington, 1977). Additionally, patients with selective impairment of auditory STM often showed a severe reduction in multiple word or sentence repetition abilities, whereas their abilities of single word repetition were relatively spared (Warrington et al., 1971). Together with these findings, our data suggest that sentence repetition and auditory STM are related.

### 4.2 Neural substrates of auditory STM

Our study identified neural structures in the left temporo-parietal areas to be involved in digit span, consistent with previous lesional and functional imaging studies in healthy participants (Baldo et al., 2012; Warrington et al., 1971; Becker et al., 1999). Although it was well recognized that the digit span task may be affected by linguistic impairment previous studies did not rule out this effect, instead taking the results directly as the representative of auditory STM ability (Martin and Ayala, 2004). Baldo used word span and digit span to assess auditory STM; however, they could not take other factors involving linguistic impact into account (Baldo et al., 2012). Therefore, speech production, perception, and executive function were included in our study as covariates according to Leff’s study (Leff et al., 2009). We found that the damage to the left SMG, IPL and AF was negatively correlated with corrected digit span tasks by controlling for demographic, lesional and relevant language variables, implying that the integrity of these brain regions supports auditory-STM capacity. As mentioned above, the phonological loop is the core component of STM, and functional imaging studies have suggested that it is located in the left IPL (Buchsbaum and D'Esposito, 2008). Papagno proposed a schematic map of the phonological loop, including relevant cortical and subcortical regions, including AF (Papagno et al., 2017). Besides, a direct electrical stimulation study indicated that the left parietal cortex and AF could play roles in auditory STM (Poeppel, 1996; Romero et al., 2006). Moreover, VLSM results demonstrated that digit span scores were associated with lesions in both the left SMG and the superior–posterior temporal areas after glioma removal (Pisoni et al., 2019). Nevertheless, evidence has shown that the left posterior STG is implicated as a common region associated with auditory STM and speech comprehension (Leff et al., 2009). According to the dual-stream model, left posterior STG is of great significance in phonological maintenance processing, but most studies draw this conclusion from backward digit span tasks and word or non-word repetition tasks. The VBM study has demonstrated that the density of the left posterior STG can predict auditory STM ability in healthy adults, with auditory STM ability evaluated using a combination of forward and backward digit span and the Spoonerisms test (Richardson et al., 2011). During serial order processing, the left IPL may play a critical role, whereas the left posterior STG is associated with verbal maintenance of item information. Consistently, the present findings suggest that left IPL/SMG and AF are involved in STM processing, indexed by digit span tasks. After controlling for speech perception, the left posterior STG was not identified, possibly because it plays a supportive role in auditory comprehension. Furthermore, we performed partial correlation analyses and found significant correlations between auditory STM and the integrity of the above regions, even after controlling for additional lesion loads in gray matter or white matter structures. These results suggest similar contributions of gray matter and white matter structures to auditory STM (Papagno et al., 2017).

### 4.3 The left AF and IPL are associated with auditory STM and sentence repetition

Previous studies focused on whether AF was the anatomical foundation of language repetition, and confirmed their correlation (Berthier et al., 2012; Wang et al., 2020). AF is a bilateral white matter fiber tract, which connects Wernicke’s area with Broca’s area via a dorsal stream arching around the posterior perisylvian (Catani et al., 2005). Thus, AF transmits information from the temporal to the frontal cortex, as well as in the opposite direction (Matsumoto et al., 2004). Since first description by Wernicke in 1874, repetition deficits have always been attributed to lesions of AF. This point was challenged by modern clinical and neurophysiological findings indicating that AF was not necessary for repetition, although it could have a subsidiary role in it (Bernal and Ardila, 2009). Case reports found that patients with cortical lesions alone might have language deficits similar to conduction aphasia (Quigg et al., 2006), whereas lesions of AF might bring out retention of the ability to repeat (Shuren et al., 1995). These discrepancies might lie in the different hypothesis models of conduction aphasia.

Previous studies have shown that crucial regions supporting language repetition and auditory STM did not involve the left AF, raising doubts about the role of AF in auditory STM (Baldo et al., 2012). In contrast, our SVR-LSM results demonstrated an obvious overlap between brain regions associated with auditory STM capacity and sentence repetition and the left AF. This discrepancy might be explained as follows. The prior study could not investigate lesion load or AF disconnection by controlling for language variables related to digit span performance. As a matter of fact, the integrity of the AF may be affected by secondary disconnection to cortical stroke lesions, in addition to direct lesional damage. Notably, the correlations between the AF and auditory STM capacity remained significant even considering the contribution of gray matter structures to auditory STM capacity. These findings emphasize the crucial role of the AF in auditory STM capacity. The current findings support the hypothesis that auditory STM capacity is associated with sentence repetition, and that the structural integrity of the left AF is significantly correlated with both behavioral performance abilities. Additionally, our study identified the left IPL to support auditory STM capacity and sentence repetition ability. This is supported by the previous functional imaging study showing that the ability of storage during sentence processing is correlated with the activations in the left temporo-parietal regions, which are involved in digit span performance (Meyer et al., 2012).

### 4.4 Limitations

Several limitations should be noted in the present study. First, the lesions analyzed in our study included some subcortical regions, which may impact the contribution of white matter. It is expected that future studies will include a wider range of lesions to further clarify the role of white matter. Second, it has been proven that item and order storage rely on different anatomical correlates, and different memory tasks possibly reflecting auditory STM capacity are expected to be applied in future studies. Third, sentence repetition may involve other components of working memory beyond STM, such as the attention system. The relationship between language and the memory system should be further clarified by exploring the differences between STM and working memory processes. Fourth, the ceiling effects as expected in healthy controls would preclude meaningful comparisons of variance. However, the purpose of including language measures of healthy controls in this study was not to establish normative data, but to confirm the discriminant validity of the assessments. The SVR-LSM analysis was conducted exclusively in the patient cohort; thus, the ceiling effect in controls does not impact our results. Fifth, considering the marginally significant association of the AF lesion load with sentence repetition and a lack of analysis of the subdividing segments of the AF, the specific tract-behavior correlations should be interpreted with caution. Additionally, post-stroke chronicity varied widely among patients. Although chronicity was included as an additional covariate in the relevant analyses, neuroplasticity and spontaneous recovery might potentially modulate the lesion-behavior relationships at different stages post-stroke. Finally, given the cross-sectional nature of the present data, the left AF and IPL are implicated in auditory STM and sentence repetition performance after chronic left hemisphere stroke. A future longitudinal study is required to elucidate the potential causal correlates of left FA with auditory STM and repetition abilities.

## 5 Conclusion

The present study demonstrated a tight correlation between auditory STM capacity and sentence repetition among chronic stroke patients with aphasia. The AF and IPL in the left hemisphere are associated with auditory STM capacity and sentence repetition following left hemisphere stroke.

## Data Availability

The raw data supporting the conclusions of this article will be made available by the authors, without undue reservation.

## Footnotes

1
https://www.nitrc.org/projects/mricron

2
http://www.neuro.uni-jena.de/cat/

3
http://www.fmrib.ox.ac.uk/fsl/

4
http://www.nitrc.org/projects/dtitk/
